# Supplementary material for: Allopurinol and blood pressure variability following ischemic stroke and transient ischemic attack: a secondary analysis of XILO-FIST
Source: J Hum Hypertens. 2024 Mar 4;38(4):307–13. doi: 10.1038/s41371-024-00906-5 (PMC11001576; doi:10.1038/s41371-024-00906-5)
Supplement: Supplementary file 1 — Supplementary Material [file 41371_2024_906_MOESM1_ESM.docx]

**Allopurinol and Blood Pressure Variability**

**Supplementary Material**

**Contents**

- **Table S1**…………………………………………………………………………….1
- **Table S2**…………………………………………………………………………….3
- **Table S3**…………………………………………………………………………….5
- **Table S4**…………………………………………………………………………….6
- **Table S5**…………………………………………………………………………….7
- **Table S6**………………………………………………………………………….…8
- **Table S7**………………………………………………………………………........9
- **Table S8**………………………………………………………………………...…11
- **Table S9**……………………………………………………………………….......12
- **Table S10**……………………………………………………………………….....13

**Table S1 – Full Inclusion and Exclusion Criteria**

| **Inclusion Criteria** | **Exclusion Criteria** |
| --- | --- |
| - Ischaemic stroke/ischaemic lesion on brain imaging in relevant anatomical territory in patients with transient ischaemic attack - Age greater than 50 years - Consent within one month of stroke | - Modified Rankin Scale score of 5 - Diagnosis of dementia - Cognitive impairment deemed sufficient to compromise capacity or comply with the protocol - Dependent on daily help from others for basic activities prior to stroke - Significant co-morbidity or frailty likely to cause death within 24 months - Contra-indication to or indication for administration of allopurinol - Concurrent azathioprine, 6-mercaptopurine therapy, other cytotoxic therapies, cyclosporin, theophylline, and didanosine - Significant hepatic impairment - eGFR < 30 ml/min - Contraindication to MRI scanning - Women of childbearing potential - Prisoners - Active participation in another CTIMP or device trial or participation within the past month - eGFR < 60 and of Korean, Han Chinese, or Thai descent - Less than 5 clinic BP measurements after randomisation* |

*Abbreviations: MRI: magnetic resonance imaging; eGFR: estimated glomerular filtration rate; CTIMP: Clinical trial of investigational medicinal product; BP: Blood Pressure.*

* <5 clinic BP measurements after randomisation is an exclusion criterion of the present analysis. All other criteria refer to eligibility for the XILO-FIST trial[19].

**Table S2 – Common Blood Pressure Variability Indices**

| **BPV Index** | **Formula** | **Description** |
| --- | --- | --- |
| Range | $Range={BP}_{max}-{BP}_{min}$ | Whilst a simple measure of variability, given its dependency on only 2 values, range is an unstable measure and can be heavily influenced by a single outlier. |
| Standard Deviation  (SD) | $SD= \sqrt{\left( \frac{1}{n-1} \right)\sum_{i=1}^{n} \left( {BP}_{i}-{BP}_{mean} \right)^{2}}$ | SD quantifies the dispersion of BP readings from the mean but ignores the order of the measurements and is typically highly correlated with the mean. |
| Average Real Variability  (ARV) | $ARV=\left( \frac{1}{n-1} \right)\sum_{i=1}^{n-1} \left\vert{BP}_{i+1}-{BP}_{i} \right\vert$ | By calculating the average of the absolute changes between consecutive readings, ARV accounts for the sequence of the BP measurements[2]. |
| Coefficient of Variation  (CV) | $CV=\left( \frac{SD}{{BP}_{mean}} \right)\times100$ | A method to correct the correlation of BPV estimates with mean BP levels is to calculate the CV. However, a weak correlation with mean BP tends to remain. |
| Variation Independent of the Mean  (VIM) | $VIM= \frac{SD}{{{BP}_{mean}}^{p}}\times\mu^{p}$  *μ* = mean BP of population  *p* derived by curve fitting | VIM is a transformation of SD uncorrelated with mean BP levels[4]. *p* is derived by fitting a curve using the model $SD=k{{BP}_{mean}}^{p}$ through a plot of SD against mean BP for all individuals within the cohort. By setting *k* and *p* as parameters, *p* can then be obtained through non-linear regression. *p* is specific to the cohort used in its estimation and thus VIM cannot be compared across different populations. |

*Abbreviations: BPV: Blood Pressure Variability; SD: Standard Deviation; BP: Blood Pressure; ARV: Average Real Variability; CV: Coefficient of Variation; VIM: Variation Independent of the Mean.*

**Table S3 - Short-term (ABPM) Blood Pressure Variability at Baseline**

| **BP Parameter** | **Placebo**  n = 100, mean ± SD | **Allopurinol**  n = 96, mean ± SD | **Between Group Difference**  (95% CI) | **P value** |
| --- | --- | --- | --- | --- |
| SBP_Mean_ (mmHg) | 125.0 ± 12.4 | 125.1 ± 13.9 | -0.15 (-3.85, 3.55) | 0.935 |
| SBP_Range_ (mmHg) | 57.1 ± 13.2 | 58.4 ± 14.7 | -1.27 (-5.21, 2.66) | 0.524 |
| SBP_SD_ (mmHg) | 14.1 ± 3.5 | 14.4 ± 3.8 | -0.33 (-1.35, 0.68) | 0.518 |
| SBP_ARV_ (mmHg) | 10.9 ± 2.3 | 11.5 ± 2.9 | -0.61 (-1.42, 0.20) | 0.142 |
| SBP_CV_ (%) | 11.3 ± 2.9 | 11.5 ± 2.9 | -0.22 (-1.03, 0.59) | 0.597 |
| SBP_VIM_ (units) | 14.1 ± 3.5 | 14.4 ± 3.6 | -0.30 (-1.29, 0.70) | 0.559 |
| DBP_Mean_ (mmHg) | 73.9 ± 7.3 | 72.8 ± 7.8 | 1.15 (-0.99, 3.29) | 0.292 |
| DBP_Range_ (mmHg) | 42.0 ± 10.0 | 40.9 ± 11.2 | 1.10 (-1.88, 4.09) | 0.467 |
| DBP_SD_ (mmHg) | 10.2 ± 2.6 | 9.9 ± 2.5 | 0.28 (-0.43, 1.00) | 0.436 |
| DBP_ARV_ (mmHg) | 8.2 ± 2.0 | 8.3 ± 2.3 | -0.04 (-0.68, 0.61) | 0. 908 |
| DBP_CV_ (%) | 13.8 ± 3.5 | 13.6 ± 3.3 | 0.21 (-0.75, 1.17) | 0. 673 |
| DBP_VIM_ (units) | 10.2 ± 2.5 | 9.9 ± 2.4 | 0.21 (-0.49, 0.90) | 0.560 |

N = 196. Differences are given as placebo subtract allopurinol (a positive difference denotes a change in favour of allopurinol). Participants had a mean of 30.3±8.2 (21.8±7.1 daytime and 8.5±2.1 night-time) BP readings taken during ABPM at baseline.

*Abbreviations: BP = Blood Pressure; SBP = Systolic BP; DBP = Diastolic BP; n = number; SD = Standard Deviation; CI = Confidence Interval; ARV = Average Real Variability; CV = Coefficient of Variation; VIM = Variation Independent of Mean.*

**Table S4 – Baseline Characteristics**

(See separate Excel file)

Baseline demographics are compared between the included and excluded cohorts. A bold p-value indicates statistical significance (<0.05).

*Abbreviations: N: Number; SD: Standard Deviation; BMI: Body Mass Index; TIA: Transient Ischaemic Attack; MI: Myocardial Infarction; PAD: Peripheral Arterial Disease; NIHSS: National Institute of Health Stroke Scale; MRS: Modified Rankin Scale; SBP: Systolic Blood Pressure; DBP: Diastolic Blood Pressure; ABPM: Ambulatory Blood Pressure Monitoring; eGFR: Estimated Glomerular Filtration Rate; MoCA: Montreal Cognitive Assessment.*

**Table S5 – Visit-to-Visit (Clinic) Blood Pressure Variability Outcomes in People with Hyperuricaemia**

| **BP Parameter** | **Placebo**  n = 29,  mean ± SD | **Allopurinol**  n = 33,  mean ± SD | **Unadjusted Between Group Difference**  (95% CI) | **P value** |
| --- | --- | --- | --- | --- |
| SBP_Range_ (mmHg) | 32.3 ± 12.1 | 35.1 ± 14.2 | -2.86 (-9.52, 3.80) | 0.394 |
| SBP_SD_ (mmHg) | 12.4 ± 4.8 | 13.3 ± 5.1 | -0.98 (-3.48,1.53) | 0.440 |
| SBP_ARV_ (mmHg) | 13.7 ± 5.3 | 14.8 ± 6.1 | -1.07 (-3.96, 1.83) | 0.464 |
| SBP_CV_ (%) | 9.2 ± 3.5 | 9.5 ± 3.5 | -0.35 (-2.12, 1.41) | 0.691 |
| SBP_VIM_ (units) | 12.7 ± 4.8 | 12.9 ± 4.7 | -0.12 (-2.54, 2.29) | 0.919 |
| DBP_Range_ (mmHg) | 17.7 ± 8.7 | 19.7 ± 7.1 | -2.02 (-6.10, 2.05) | 0.324 |
| DBP_SD_ (mmHg) | 6.7 ± 3.3 | 7.4 ± 2.5 | -0.72 (-2.24, 0.80) | 0.343 |
| DBP_ARV_ (mmHg) | 7.1 ± 3.4 | 8.5 ± 2.9 | -1.38 (-3.00, 0.24) | 0.093 |
| DBP_CV_ (%) | 8.8 ± 4.6 | 9.3 ± 3.2 | -0.51 (-2.55, 1.53) | 0.618 |
| DBP_VIM_ (units) | 6.8 ± 3.4 | 7.4 ± 2.5 | -0.61 (-2.15, 0.94) | 0.434 |

N = 62. Differences are given as placebo subtract allopurinol (a positive difference denotes a change in favour of allopurinol).

*Abbreviations: BP = Blood Pressure; SBP = Systolic BP; DBP = Diastolic BP; n = number; SD = Standard Deviation; CI = Confidence Interval; ARV = Average Real Variability; CV = Coefficient of Variation; VIM = Variation Independent of Mean*

**Table S6 – Visit-to-Visit (Clinic) Blood Pressure Variability Outcomes in People without Hyperuricaemia**

| **BP Parameter** | **Placebo**  n = 130,  mean ± SD | **Allopurinol**  n = 123,  mean ± SD | **Unadjusted Between Group Difference**  (95% CI) | **P value** |
| --- | --- | --- | --- | --- |
| SBP_Range_ (mmHg) | 31.5 ± 14.1 | 29.9 ± 12.9 | 1.62 (-1.72, 4.97) | 0.341 |
| SBP_SD_ (mmHg) | 11.8 ± 5.3 | 11.4 ± 5.0 | 0.43 (-0.85, 1.71) | 0.510 |
| SBP_ARV_ (mmHg) | 12.9 ± 6.2 | 12.6 ± 6.3 | 0.22 (-1.33, 1.77) | 0.783 |
| SBP_CV_ (%) | 8.5 ± 3.5 | 8.4 ± 3.5 | 0.09 (-0.79, 0.96) | 0.848 |
| SBP_VIM_ (units) | 11.4 ± 4.7 | 11.5 ± 4.9 | -0.11 (-1.29, 1.08) | 0.857 |
| DBP_Range_ (mmHg) | 18.4 ± 8.4 | 18.9 ± 8.2 | 0.53 (-1.54, 2.59) | 0.616 |
| DBP_SD_ (mmHg) | 7.0 ± 3.1 | 6.8 ± 3.0 | 0.18 (-0.57, 0.93) | 0.638 |
| DBP_ARV_ (mmHg) | 7.8 ± 3.8 | 7.7 ± 3.5 | 0.06 (-0.84, 0.96) | 0.895 |
| DBP_CV_ (%) | 8.7 ± 3.8 | 8.6 ± 3.9 | 0.12 (-0.83, 1.07) | 0.804 |
| DBP_VIM_ (units) | 6.9 ± 3.0 | 6.8 ± 3.0 | 0.15 (-0.60, 0.89) | 0.696 |

N = 253. Differences are given as placebo subtract allopurinol (a positive difference denotes a change in favour of allopurinol).

*Abbreviations: BP = Blood Pressure; SBP = Systolic BP; DBP = Diastolic BP; n = number; SD = Standard Deviation; CI = Confidence Interval; ARV = Average Real Variability; CV = Coefficient of Variation; VIM = Variation Independent of Mean*

**Table S7 – Change in Short-term (ABPM) Blood Pressure Variability from Baseline to Week 104**

| **BP Parameter** | **Change with Placebo**  n =61,  mean ± SD | **Change with Allopurinol**  n = 54,  mean ± SD | **Unadjusted Between Group Difference**  (95% CI) | **P value** | **Adjusted Between Group Difference**  (95% CI) | **P value** |
| --- | --- | --- | --- | --- | --- | --- |
| SBP_Mean_ (mmHg) | -0.9 ± 11.0 | -5.20 ± 13.1 | 4.33 (-0.18, 8.85) | 0.060 |  |  |
| SBP_Range_ (mmHg) | -0.9 ± 17.4 | -1.2 ± 19.4 | 0.28 (-6.53, 7.09) | 0.934 | -1.49 (-8.18, 5.21) | 0.661 |
| SBP_SD_ (mmHg) | 0.5 ± 4.7 | 0.0 ± 4.2 | 0.57 (-1.08, 2.22) | 0.495 | 0.16 (-1.46, 1.79) | 0.844 |
| SBP_ARV_ (mmHg) | 0.1 ± 2.6 | -0.1 ±3.7 | 0.21 (-1.01, 1.43) | 0.738 | -0.15 (-1.36, 1.07) | 0.813 |
| SBP_CV_ (%) | 0.5 ± 3.8 | 0.5 ± 3.0 | -0.01 (-1.30, 1.27) | 0.983 | 0.08 (-1.23, 1.39) | 0.905 |
| SBP_VIM_ (units) | 0.5 ± 4.6 | 0.2 ± 3.9 | 0.25 (-1.35, 1.84) | 0.760 | 0.11 (-1.51, 1.73) | 0.898 |
| DBP_Mean_ (mmHg) | -1.8 ± 5.5 | -2.3 ± 7.3 | 0.47 (-1.90, 2.85) | 0.693 |  |  |
| DBP_Range_ (mmHg) | -2.8 ± 11.8 | -0.1 ± 16.6 | -2.71 (-7.99, 2.58) | 0.313 | -2.94 (-8.12, 2.25) | 0.265 |
| DBP_SD_ (mmHg) | -0.2 ± 2.9 | -0.2 ± 3.4 | 0.00 (-1.16, 1.17) | 0.994 | -0.05 (-1.19, 1.09) | 0.933 |
| DBP_ARV_ (mmHg) | -0.6 ± 2.2 | -0.3 ± 2.8 | -0.28 (-1.25, 0.69) | 0.571 | -0.29 (-1.27, 0.69) | 0.557 |
| DBP_CV_ (%) | 0.0 ± 4.2 | 0.1 ± 4.1 | -0.09 (-1.63, 1.46) | 0.911 | -0.08 (-1.63, 1.48) | 0.923 |
| DBP_VIM_ (units) | -0.2 ± 3.0 | -0.1 ± 3.1 | -0.04 (-1.17, 1.09) | 0.942 | -0.06 (-1.19, 1.08) | 0.920 |

N = 115. Changes were calculated as visit subtract baseline. Between group differences are given as placebo subtract allopurinol (a positive difference denotes a change in favour of allopurinol). Analyses were adjusted for the change in mean systolic or diastolic BP.

*Abbreviations: BP = Blood Pressure; SBP = Systolic BP; DBP = Diastolic BP; n = number; SD = Standard Deviation; CI = Confidence Interval; ARV = Average Real Variability; CV = Coefficient of Variation; VIM = Variation Independent of Mean.*

**Table S8 – Correlation Analysis of Visit-to-Visit Blood Pressure Variability and Change in White Matter Hyperintensity Volume**

| **BPV Measure** | **Correlation Coefficient (r)** | **95% CI for r** | **P value** |
| --- | --- | --- | --- |
| SBP_Range_ | 0.06 | -0.04, 0.16 | 0.230 |
| SBP_SD_ | 0.08 | -0.02, 0.18 | 0.126 |
| SBP_ARV_ | 0.09 | -0.01, 0.19 | 0.082 |
| SBP_CV_ | 0.06 | -0.05, 0.16 | 0.276 |
| SBP_VIM_ | 0.04 | -0.06, 0.14 | 0.463 |
| DBP_Range_ | 0.06 | -0.04, 0.16 | 0.239 |
| DBP_SD_ | 0.07 | -0.04, 0.17 | 0.210 |
| DBP_ARV_ | 0.07 | -0.03, 0.17 | 0.162 |
| DBP_CV_ | 0.05 | -0.05, 0.16 | 0.304 |
| DBP_VIM_ | 0.06 | -0.04, 0.16 | 0.241 |

N = 367. *Abbreviations: BPV = Blood Pressure Variability; CI = Confidence Interval; SBP = Systolic BP; DBP = Diastolic BP; SD = Standard Deviation; ARV = Average Real Variability; CV = Coefficient of Variation; VIM = Variation Independent of Mean*

**Table S9 – Correlation Analysis of Visit-to-Visit Blood Pressure Variability and Change in Brain Volume**

| **BPV Measure** | **Correlation Coefficient (r)** | **95% CI for r** | **P value** |
| --- | --- | --- | --- |
| SBP_Range_ | 0.02 | -0.08, 0.12 | 0.710 |
| SBP_SD_ | 0.01 | -0.09, 0.12 | 0.785 |
| SBP_ARV_ | 0.04 | -0.06, 0.15 | 0.410 |
| SBP_CV_ | 0.01 | -0.10, 0.11 | 0.926 |
| SBP_VIM_ | -0.01 | -0.11, 0.10 | 0.933 |
| DBP_Range_ | -0.08 | -0.18, 0.03 | 0.138 |
| DBP_SD_ | -0.10 | -0.20, 0.01 | 0.068 |
| DBP_ARV_ | -0.05 | -0.15, 0.06 | 0.354 |
| DBP_CV_ | -0.09 | -0.19, 0.02 | 0.108 |
| DBP_VIM_ | -0.09 | -0.19, 0.01 | 0.079 |

N = 359. *Abbreviations: BPV = Blood Pressure Variability; CI = Confidence Interval; SBP = Systolic BP; DBP = Diastolic BP; SD = Standard Deviation; ARV = Average Real Variability; CV = Coefficient of Variation; VIM = Variation Independent of Mean*

**Table S10 – Correlation Analysis of Visit-to-Visit Blood Pressure Variability and Change in Cognitive Function**

| **BPV Measure** | **Correlation Coefficient (r)** | **95% CI for r** | **P value** |
| --- | --- | --- | --- |
| SBP_Range_ | -0.01 | -0.12, 0.09 | 0.793 |
| SBP_SD_ | -0.02 | -0.12, 0.08 | 0.719 |
| SBP_ARV_ | -0.03 | -0.13, 0.07 | 0.570 |
| SBP_CV_ | -0.01 | -0.11, 0.09 | 0.872 |
| SBP_VIM_ | -0.01 | -0.10, 0.10 | 0.986 |
| DBP_Range_ | 0.02 | -0.08, 0.12 | 0.729 |
| DBP_SD_ | 0.04 | -0.07, 0.14 | 0.489 |
| DBP_ARV_ | 0.07 | -0.04, 0.17 | 0.202 |
| DBP_CV_ | 0.02 | -0.08, 0.12 | 0.723 |
| DBP_VIM_ | 0.03 | -0.07, 0.13 | 0.572 |

N = 368. *Abbreviations: BPV = Blood Pressure Variability; CI = Confidence Interval; SBP = Systolic BP; DBP = Diastolic BP; SD = Standard Deviation; ARV = Average Real Variability; CV = Coefficient of Variation; VIM = Variation Independent of Mean*
